# Hypoxia and therapeutic treatment of EV-A71 with an immune modulator TLR7 agonist in a new immunocompetent mouse model

**DOI:** 10.1186/s12929-019-0585-y

**Published:** 2019-11-11

**Authors:** An-Ting Liou, Chun-Che Liao, Shu-Fan Chou, Ya-Shu Chang, Chih-Shin Chang, Chiaho Shih

**Affiliations:** 1 Institute of Biomedical Sciences, Academia Sinica, Taipei, Taiwan; 2 Institute of Microbiology and Immunology, National Yang-Ming University, Taipei, Taiwan

**Keywords:** EV-A71, enterovirus, hypoxia, White-Jade muscle, therapy, mouse model

## Abstract

**Background:**

Enterovirus 71 (EV71 or EV-A71) was first identified in California about half a century ago. In recent years, outbreaks of EV-A71 were prevalent worldwide, including Taiwan, Malaysia, Singapore, Japan, and China. Between 2008 and 2011, China alone reported 1894 deaths associated with EV-A71 infection. In mild cases, EV-A71 can cause herpangina and hand-foot-and-mouth disease (HFMD). However, in severe cases, it could cause neurological disorders, including meningitis and encephalitis. Cardiopulmonary failure is common among hospitalized children with EV-A71 infection. No effective FDA-approved therapeutics against EV-A71 are clinically available.

**Methods:**

We report the establishment of an immunocompetent wild type strain 129 (wt-129) mouse model, which can be cross-species infected with human EV-A71 clinical isolates via an intraperitoneal route.

**Results:**

One intriguing disease phenotype of this new model is the development of characteristic “White-Jade” patches in the muscle, which lost sporadically the normal pink color of uninfected muscle. Viral VP1 protein and massive leukocyte infiltration were detected in muscles with or without white-jades. We demonstrated further that hypoxia is a general phenomenon associated with white-jades in both immunocompetent and immunodeficient mouse models. Therefore, hypoxia appears to be a feature intrinsic to EV-A71 infection, irrespective of its host’s immunogenetic background. To date, no effective treatment for EV-A71 is available. Here, using this new wt-129 mouse model, we showed that timely treatment with compound R837 (a TLR7 immune modulator) via oral or intraperitoneal routes, rescued the hypoxia, limb paralysis, and death at a high therapeutic efficacy.

**Conclusions:**

In this new immunocompetent mouse 129 model, we observed an unexpected white-jade phenotype and its associated hypoxia. The successful treatment with TLR7 immune modulators via an oral route, provide us a new research direction for EV-A71 basic science and translational research. It remains an open issue whether R837 or its related compounds, will be a promising drug candidate in clinical trials in EV-A71 endemic or epidemic areas in the future.

## Background

Enterovirus 71 (EV-A71) was first isolated in California in 1969 [1, 2]. As a member of *Picornaviridae*, EV-A71 is close to poliovirus [3, 4], coxsakie virus [5], and hepatitis A virus (EV72) [6]. In mild cases, EV-A71 causes hand-foot-and-mouth disease (HFMD), herpangina and diarrhea in children [1, 2, 7]. However, in severe cases, it can cause encephalitis, cardiopulmonary failure, and death [8, 9]. EV-A71 infection is a major health threat worldwide, including the Asia-Pacific region [7, 10], USA [11], Europe [12, 13] and Russia [14].

Identification of human receptors of EV-A71 was reported in 2009. Human scavenger receptor class B (hSCARB2) was found to bind with EV-A71 and support EV-A71 infection in non-permissive cell lines [15]. Human P-selectin glycoprotein ligand-1 (hPSGL-1) was also found to play an important role in leukocyte infection with EV-A71 [16]. Additional potential receptors for EV-A71 include annexin II [17] and nucleolin [18]. Human SCARB2 transgenic mice were shown to be susceptible to EV-A71 infection [19, 20]. However, these hSCARB2 transgenic mouse models are not without any limitations in research. For example, in one report [20], the hSCARB2 transgenic model was only 2-fold more susceptible to EV-A71 infection (genotype B) than the wild type mouse C57BL/6. In the other report [19], the transgenic model is particularly susceptible to a so-called Isehara strain of EV-A71 in Japan. Both transgenic models have very low oral infection efficiency.

In addition to the receptor transgenic models, certain strains of immunodeficient mice are alternative models for EV-A71 in vivo research, such as strains AG129 [21–23], NOD/SCID [24, 25], and *stat-1* KO [24]. Most recently, we established a new hybrid mouse strain by cross-breeding between the hSCARB2 transgenic mouse and the *stat-1* KO mouse. This hybrid mouse strain developed limb paralysis with earlier onset at a much lower titer of EV-A71 genotype B or C [26]. In the case of strain AG129, it is deficient in both alpha-interferon (IFNA) and gamma-interferon (IFNG). Since this AG129 strain has been commonly used in the Zika virus research these days [27–29], it is an important issue whether the parental wild type strain 129 is also susceptible to viral infections, such as Zika virus or EV-A71 clinical isolates [21].

Oxygen consumption is essential to the bioenergetics in our life. When oxygen supply is low, hypoxia-inducible factor 1 (HIF-1) and a number of other genes will be induced in all cells [30, 31]. As a HIF-1 target, VEGF (vascular endothelial growth factor) is an important factor for angiogenesis and vascularization [32]. To date, HIF-1 and its target genes are well known to play critical roles in cancer, pulmonary diseases, stroke and coronary artery diseases, and physiological adaptation on high mountains. However, it remains largely unknown what could be the role of hypoxia in microbial infection, inflammation, pathogenesis, and recovery, if any [33].

In this study, we tested the possibility to infect the wild type immunocompetent mouse strain 129S2/SvPasCrlBltw (wt-129) with EV-A71 clinical isolates. Both IFNA and IFNG signalings can be induced by poly (I:C) or EV-A71 infection in this novel mouse model. Approximately 43% of infected mice developed limb paralysis and death. Unexpectedly, about 50% of these paralyzed mice exhibited a “White-Jade” muscle phenotype. This phenotype is characterized by its locally discolored and sometimes with fibrosis patches, over the pink-colored background muscle. Viral VP1 protein can be detected in the spinal cord and muscle of infected wt-129 mice by immunohistochemical (IHC) staining. Regeneration and massive leukocyte infiltration were detected in the muscle. To date, no FDA-approved antivirals for EV-A71 is available. Using this wt-129 model for therapeutic studies, we successfully protected EV-A71-infected mice from paralysis and death by administering mouse IFNα or a TLR7 agonist R837 in a timely manner. Although another compound GS9620 could also protect the infected mice [34], it caused a very severe side effect of diarrhea in this mouse 129 model. In conclusion, this wild type strain 129 mice can serve as an immunocompetent mouse model for EV-A71 pathogenesis and pre-clinical research. It also warrants further investigation whether anti-hypoxia could be a therapeutic strategy against EV-A71 infection and pathogenesis.

## Materials and methods

### Ethics statement

All animal experiments were conducted under protocols approved by Academia Sinica Institutional Animal Care & Utilization Committee (ASIACUC Protocol number 13–12-622). Research was conducted in compliance with the principles stated in the Guide for the Care and Use of Laboratory Animals, National Research Council, 1996.

### Experimental infection

All mice were housed under specific-pathogen-free conditions in individual ventilated cages. NOD/SCID and strain 129S2/SvPasCrlBltw (abbreviated as wt-129) mice were purchased from BioLASCO (Taiwan). One-week-old mice were infected with EV-A71 at the dose of 10^7^–10^8^ pfu/mouse via the intraperitoneal route. Disease manifestations were monitored daily post-infection.

### Polyinosinic-polycytidylic acid stimulation in the mouse model

Three-week-old wt-129 mice were challenged with low molecular weight (Poly (I:C)-LMW) (thrl-picw, InvivoGen) and high molecular weight (Poly (I:C)-HMW) (thrl-pic, InvivoGen) at the dose 10 μg/g of mice. Sera were collected at 3 and 6 h post-inoculation with Poly (I:C) for the ELISA assay of mouse IFNA (Cat. No. 42120, PBL Assay Science; Cat. No. BMS6028, Affymetrix).

### Treatment with interferon signaling

Neutralizing antibodies against mouse IFN- alpha receptor or IFN- gamma (Bio X Cell) were i.p. injected at a dose of 150 μg/mouse into 1-week-old wt-129 mice, either before or after i.p. inoculation with EV-A71 clinical isolate F23 (10^8^ PFU/mouse). The neutralizing antibody against the IFN-alpha receptor was injected 1 day before EV-A71 inoculation, followed by repetitive antibody treatments on 1 and 3 dpi. Recombinant mouse IL28A and IL28B were both purchased from R & D Systems®. Various doses of mouse IL28 was i.p. injected into mice at dpi = 1 (Additional file 4: Figure S4a). Recombinant mouse IFNαA (PBL Assay Science Co.), GS9620 (Cayman Chemical) and R837 (InvivoGen), were orally or i.p. injected into wt-129 mice infected with EV-A71, at the dose of 300 IU/g (mouse IFNαA), 3.0 μg/g (GS-9620), 4.5μg/g (GS-9620), and 5.0 μg/g (R837), respectively.

### Generation of IL28b KO mouse model

Two IFN-lambda knockout (KO) mouse lineages were established in this study. Briefly, ES cells containing IL-28b knockout on mouse chromosome 7 from C57BL/6 N (black) mice were purchased from KOMP Repository, UC Davis, California, USA. ES cells with IL-28b knockout was generated by homologous recombination using a reporter cassette ZEN-UB1. The resulting deletion starts at 28,522,821, and ends at 28,524,338. Genetically modified ES cells were microinjected into the blastocysts of C57BL/6 J (white) (Transgenic Core Facility, Academia Sinica, Taiwan). Coat color chimeric mice were generated as F0, and then backcrossed with parental C57BL/6 N (black) mice. Genotypings were conducted by real time PCR. Homozygous KO mice exhibited no PCR signals, while both wild type and heterozygote mice displayed strong signals before the 28th cycle of amplification **(**Additional file 4: Figure S4 and Additional file 6: Table S1**).**

### Virus preparation

F23 is a clinical isolate of EV-A71 (genotype B5) from a patient at the Changhua Christian Hospital, Changhua, Taiwan [24, 25]. Human rhabdomyosarcoma (RD) cells (ATCC CCL-136) were cultured in Dulbecco’s Modified Eagle medium (DMEM; Gibco) with 10% fetal bovine serum (FBS; Hyclone) and 1% penicillin-streptomycin (Gibco) at 37 °C. For virus preparation, RD cells were cultured in T175 flask with 0.2% FBS, and infected with EV-A71 at multiplicity of infection (MOI) of 0.01 at 37 °C for 24 h. Virus was harvested by three cycles of freeze-and-thaw of infected RD cells, and centrifuged at 3000×g at 4 °C for 30 min. Supernatant was concentrated by ultracentrifugation through a 30% sucrose cushion in Beckman SW28 rotors at 26000 rpm, 4 °C, for 4–6 h. EV-A71 pellets were resuspended in phosphate-buffered saline (PBS) and the viral titer was determined by plaque assy.

### Virus titration

RD cell monolayer was cultured at a density around 5 × 10^5^ cells/well in 6-well plates (SPL life science). EV-A71 stock was 10-fold serially diluted with DMEM, and RD cells were infected with virus at various dilutions. After 1 h incubation, virus was removed from RD cells, and the cell monolayer was covered with 4 mL DMEM containing 0.3% low-melt agarose (Lonza) and 0.2% FBS at 37 °C for 72 h. After 72 h, RD cells were fixed with 3.7% formalin (Merck) at room temperature for 1 h, and the number of plaques was scored after crystal violet staining.

### Complete blood count (CBC) analysis

The blood samples were collected by submandibular blood collection, and 1.5 g/dL of Na_2_EDTA was used as anticoagulant. The whole blood was analyzed by IDEXX ProCyte Dx®Hematology Analyzer. (Taiwan Mouse Clinic).

### VEGFA ELISA assay

Mouse sera and muscle samples were collected from EV-A71 infected mice after disease manifestation. The muscle samples were homogenized with PBS at 0.1 mg/uL. Supernatant was concentrated by centrifugation at 12000 rpm, 4 °C. The expression level of VEGFA was measured according to the manufacturer’s instruction (Cat. No. BMS619/2, Affymetrix).

### Histopathological and immunohistochemical (IHC) stain

The euthanized mice were perfused transcardially with PBS, followed by 10% Neutral Buffered Formalin (CHIN I PAO CO., Taiwan). Tissue blocks were fixed in 10% neutral buffered formalin overnight. Fixed tissues were paraffin embedded, sliced, and stained with H & E by the Pathology Core Laboratory, IBMS, Academia Sinica, Taiwan. Xylene and ethanol were used for deparaffinization and rehydration. For antigen retrieval and enzyme blocking, retrieval buffer pH 6.0 (Dako) and endogenous enzyme blocker (Dako) were used. Slides were washed with PBS containing 0.1% Tween 20 (PBST), followed by incubation with specific antibodies, including rabbit anti-VP1 polyclonal antibody (PB7631, Abnova, Taiwan), rabbit anti-HIF1A polyclonal antibody (NB100–479, Novus), rabbit anti-VEGFA polyclonal antibody (ab46154, Abcam), rabbit anti-myogenin monoclonal antibody (GTX 63352, Genetex, Taiwan), as well as antibodies specific for the immune system, CD3 (MA1–90582, Thermo), CD19 (PAB19567, Abnova), CD68 (ab955, Abcam), and CD163 (bs-2527, Bioss), respectively. These slides were washed with PBS and incubated with anti-rabbit secondary antibody. DAB system (Dako) was used to visualize signals of antigens. Sectioned samples were counterstained with haematoxylin (J.T. Baker), and mounted with mounting reagent (MUTO Pure Chemicals). Images were scanned and presented via Pannoramic 250 FLASH (3DHISTECH Ltd).

### Statistical analysis

Clinical scores of experimental mice were analyzed by one-way ANOVA. Survival rates of infected mice were analyzed with the Log-rank test. Paralysis rates and cytokine expression experiments were analyzed by Student’s *t* test. *P < 0.05; **P < 0.01; ***P < 0.001.

## Results

### A new immunocompetent wild type 129 mouse model for EV-A71 infection

**Fig. 1:**
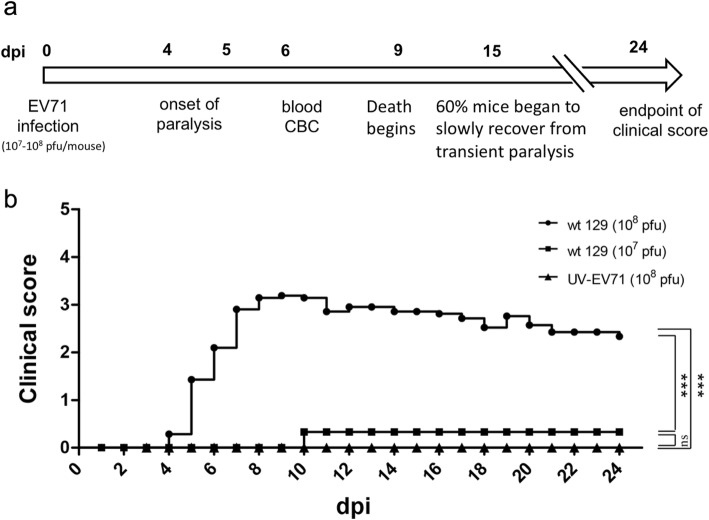
Infection and pathogenesis with clinical isolates of EV-A71 in wild type strain 129 mice. **a** An illustration of the experimental design and time course using a wt-129 mouse model. **b** Disease and death were detected in the immunocompetent wild type mouse strain 129 experimentally infected with EV-A71. One-week-old newborns of wild type 129 mice were i.p. infected with clinical isolates of EV-A71 at two different viral titers. Disease manifestation was monitored daily. Clinical scores were defined as follows: 0, healthy; 1, hair loss, wasting, or ruffled hair; 2, limb weakness; 3, paralysis in only 1 limb; 4, paralysis in 2 to 4 limbs; 5, death. No significant clinical score was observed, when inoculated with 10^7^ pfu/mouse or 10^8^ UV-inactivated virus/mouse

In EV-A71 infection experiment, one-week-old wt-129 mice were i.p. injected with clinical isolates of EV-A71 (genotype B5) at a titer of 10^7^–10^8^ pfu/mouse. Disease manifestations were monitored daily (Fig. 1a). At 10^7^ pfu/mouse, few infected mice showed limb weakness, and none showed paralysis and death. At higher titer (10^8^ pfu/mouse), infected mice first showed hindlimb weakness at dpi 4–5 and later developed limb paralysis at dpi 7 and death around dpi 9–10. The disease manifestation appeared to result from EV-A71 in vivo replication. Upon injection with UV-inactivated EV-A71 (10^8^ pfu/mouse), no disease manifestation was observed (Fig. 1b).

### A “white-jade” muscle phenotype in wt-129 mice

**Fig. 2:**
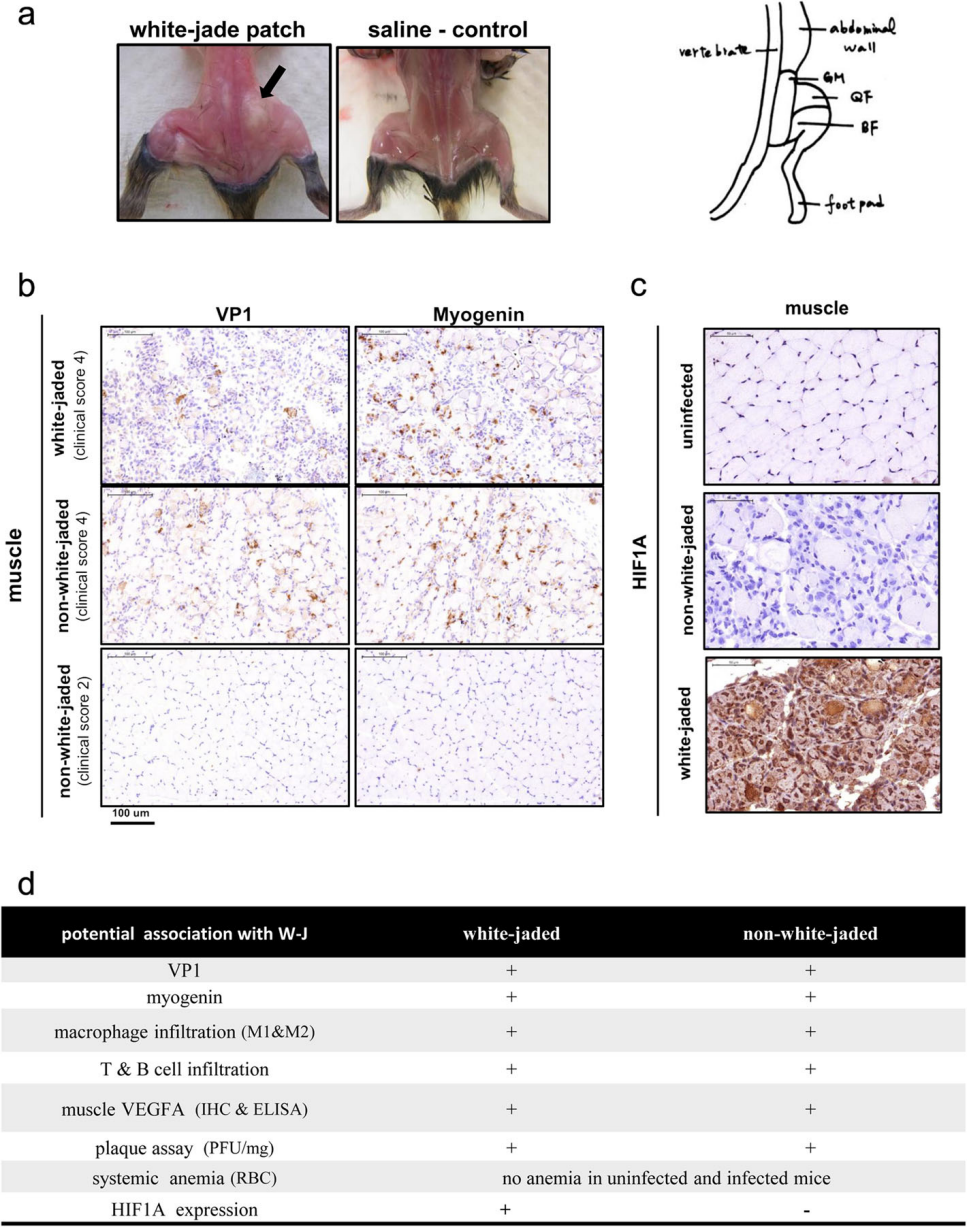
A “white-jade” muscle phenotype in EV-A71-infected mouse strain 129. **a** *Left panel*: We coined a “white-jade” phenotype to describe the pathological changes in the color of the hindlimb muscle in EV-A71-infected mice (indicated by arrow). This phenotype showed characteristic muscle with locally whitened color in appearance and hardened tissue mass. In the saline control, tendons (not muscle) can be seen in white color. *Right panel*: An anatomical sketch of the hindlimb muscle. GM: gluteal muscle, QF: quadratus femoris muscle, BF: Biceps femoris muscle. **b** Both VP1 protein (upper) and myogenin protein (lower) were detected by IHC staining in the muscles, indicating EV-A71 infection, muscle injury and regeneration. The magnification is 200X. **c** Striking expression of HIF1A (hypoxia inducible factor 1-α) protein (brown color) was detected by IHC staining in the white-jaded tissue, but not in the non-white-jaded tissue from the same mouse. **d** Specific association between HIF1A expression and white-jaded muscle

When paralyzed mice were sacrificed, we noted that their muscle tissue often displayed sporadic white patches over the normal-looking pink color background (Fig. 2a). We coined this intriguing phenotype as a “white-jade” mosaic. As shown in Fig. 2b, there is no significant difference between white-jaded and non-white-jaded muscles from diseased mice at the stage of clinical score 4 by immunohistochemical (IHC) staining with either anti-VP1 or anti-myogenin antibodies. Myogenin is a cellular transcription factor expressed during muscle regeneration [35], which is known to always follow muscle injury [36]. Both white-jaded and non-white jaded muscles showed myogenin-positive signals (right panel, Fig. 2b). Mice at the stage of clinical score 2 showed no apparent signals of VP1 or myogenin. We also found no significant difference between white-jade and non-white-jade muscles in viral load (pfu) and leukocyte infiltrations by IHC staining for lymphocyte-specific CD3 and CD19, as well as macrophage specific CD68 and CD163 (Additional file 1: Figure S1a-c).

We later reasoned that the pale color in the white-jades could result from the decreased number of red blood cells (RBC) or vascularization. We first examined the possibility of systemic anemia in EV-A71-infected mice. Complete blood count (CBC) was conducted for blood cell analysis between infected and uninfected mice. We found no apparent changes in the quality or quantity of RBC (Additional file 1: Figure S1d), including the number of red blood cells (RBC count), hemoglobin concentration (HGB value) and hematocrit (HCT value). Instead, we noted the increase of both neutrophil and monocytes in the blood, indicating an inflammatory condition in the infected mice. Next, we examined the expression of vascular endothelial growth factor (VEGF) in muscle and serum by IHC and ELISA, between infected and uninfected mice as well as between white-jades and non-white-jades. Again, we found no significant difference in VEGF expression between white-jade and non-white-jade muscles by IHC or ELISA (Additional file 1: Figure S1e and f). In contrast, statistically significant difference in VEGF expression can be detected in muscle and serum samples between infected and uninfected mice (Additional file 1: Figure S1f and g).

Since oxygen supply is mainly delivered by the hemoglobin in RBC, the pale color could reflect a locally reduced amount of hemoglobin and oxygen (hypoxia) in the white-jaded muscle. HIF1A is a master transcription factor known to be strongly expressed in a hypoxia microenvironment [37]. We compared HIF1A expression between white-jade vs. non-white-jade muscles using IHC staining with an anti-HIF1A antibody. As shown in Fig. 2c, HIF1A protein was strongly expressed in white-jaded muscle tissues, but not detectable in non-white-jaded or uninfected muscle tissues. In summary (Fig. 2d), HIF1A appears to be the most distinctive and consistent difference between white-jades and non-white-jades.

### A white-jade phenotype in NOD/SCID mice

**Fig. 3:**
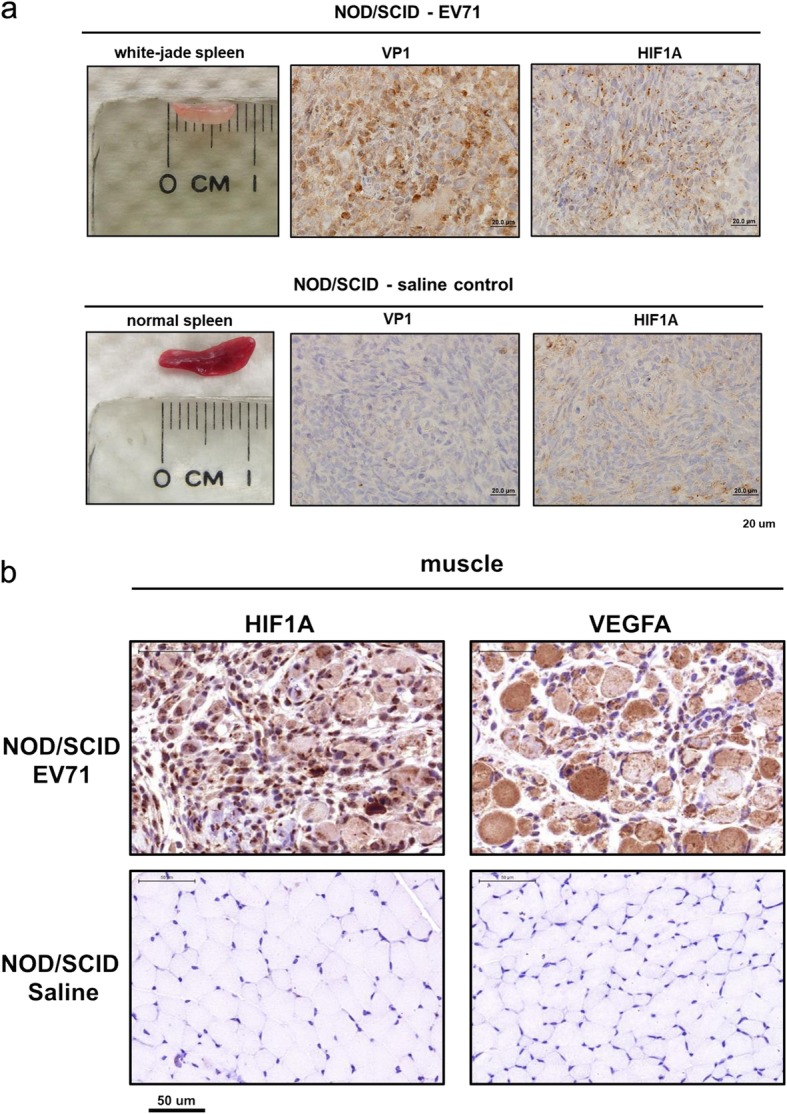
White-jaded spleen and muscle in another NOD/SCID mouse model. One-week-old NOD/SCID mice were i.p. infected with EV-A71, and scarified after disease manifestation. **a** *Upper panel***:** An atrophic and discolored spleen was identified in EV-A71-infected mice. Viral protein (VP1) and HIF1A were both expressed in the infected spleen. *Lower panel***:** A saline control showed a normal-sized spleen with no VP1 and less expression of HIF1A. **b** Both HIF1A and VEGFA were strongly expressed in the whitened muscles infected with EV-A71, but not detectable in the saline control

Is the white-jade phenotype idiosyncratic to the wt-129 model, or is it generally associated with EV-A71 pathogenesis? We addressed this issue by using an immunodeficient NOD/SCID mouse model [24]. As shown in Fig. 3, we examined the muscle and spleen sections by IHC staining for VP1 and HIF1A. Spleen atrophy was apparent in EV-A71-infected NOD/SCID mice (Fig. 3a, left panel). Interestingly, both VP1 and HIF1A proteins were highly expressed in the infected and white-jaded spleen. In addition to spleen, the completely discolored hindlimb muscle also showed strong signals of HIF1A and VEGFA (Fig. 3b). Altogether, these data indicate that hypoxia could be a rather general pathology associated with EV-A71 infection in both immunodeficient and immunocompetent mouse models (Figs. 1, 2 and 3).

### Inhibition of IFN signaling enhanced limb paralysis

**Fig. 4:**
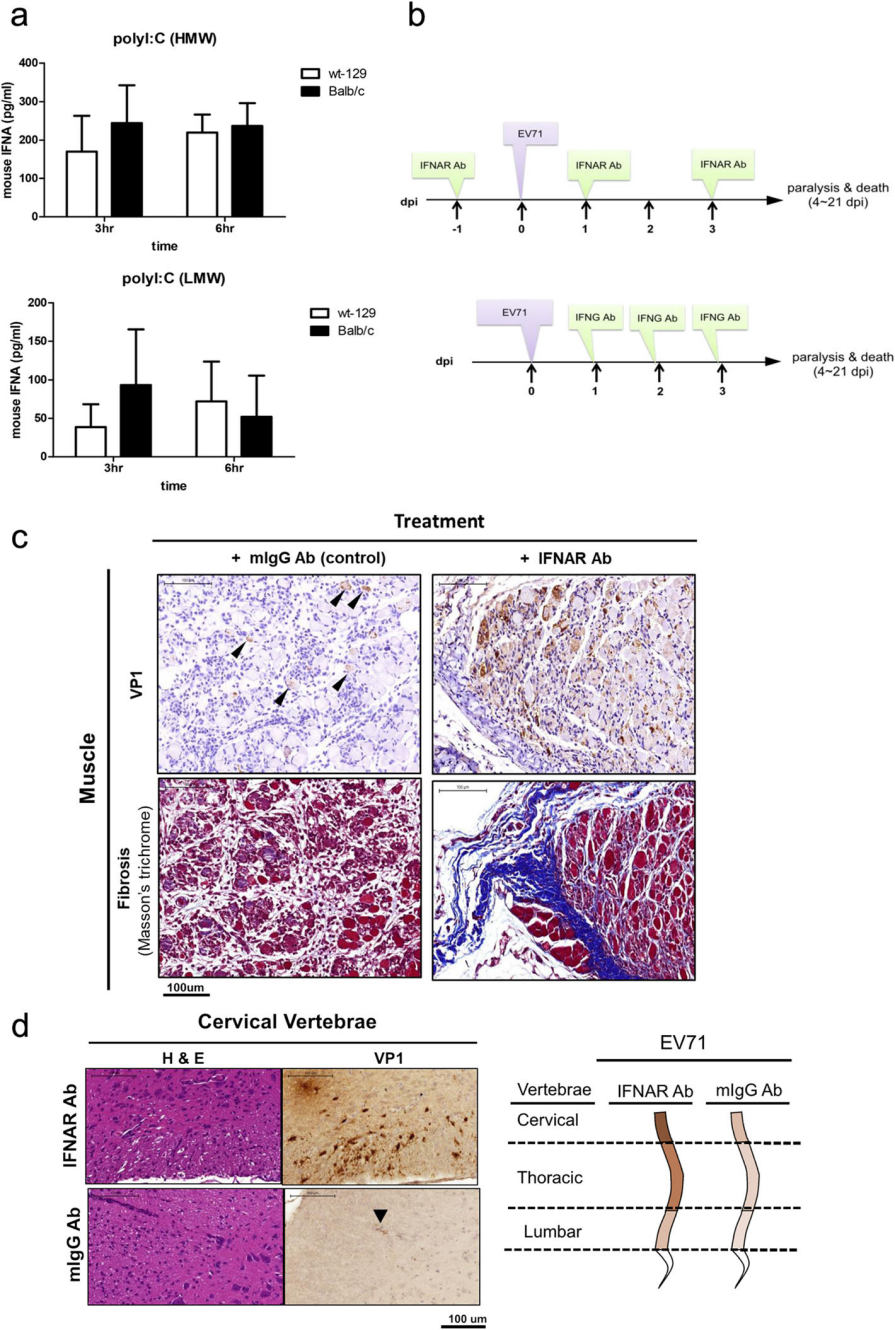
Inhibition of IFN signaling enhanced EV-A71 infection and pathogenesis. **a** Both wild type 129 and BALB/c mice were i.p. injected with high molecular weight (HMW) and low molecular weight (LMW) poly (I:C) at the dose of 10 μg/g body weight, respectively. Sera were collected at 3 h and 6 h post-injection. Mouse IFNA was measured by the ELISA assay (Materials and Methods). **b** A scheme of treatment with neutralization antibody in a wt-129 mouse model. *Upper panel*: Neutralizing antibody specific for mouse IFNAR (interferon-alpha receptor) was injected (150 μg/ mouse) into one-week-old mice via the i.p. route 1 day before infection. EV-A71 was inoculated at 10^8^ pfu/mouse, followed by two shots of IFNAR neutralizing antibody on 1 and 3 dpi. *Lower panel:* IFNG (interferon-gamma) antibody was administered on 1, 2, and 3 dpi. **c** Upon treatment with anti-IFNAR antibody, muscle fibrosis was easily detected at some white-jade patches. Sectioned white-jades from the hindlimb muscle was stained with IHC for VP1 (upper), and Masson’s trichrome staining (lower), respectively. The deep blue color reflects collagen fiber deposition and severe fibrosis in muscle. **d** *Left panel*: In mice treated with a control antibody mIgG, expression of VP1 protein in the spinal cord was only weakly detected by IHC. In mice treated with anti-IFNAR antibody, VP1 protein signal was stronger and more wide-spread in the cervical spinal cord. *Right panel:* A cartoon summary of VP1 expression in the spinal cord, including cervical, thoracic, and lumbar vertebrae

Previous studies demonstrated that EV-A71 could infect immunodeficient mice with impaired IFNA/G signaling, such as stat-1 KO or AG129 mice [21, 24]. However, IFN-α receptor knockout (*ifnar* KO) mice in C57BL/6 background were totally resistant to EV-A71 infection via several different routes [24]. To follow up on this issue, we used this new immunocompetent wild type 129 model, and asked whether IFNAR neutralizing antibody could have any effect on EV-A71 infection and pathogenesis (Fig. 4). To first validate the immune competency of the wt-129 mice obtained from vendors, we injected 3-week-old mice with low and high molecular weight poly (I:C) at the dose of 10 μg/g mouse via an intraperitoneal (i.p.) route. Sera were collected at 3 and 6 h post inoculation, and assayed for mouse interferon alpha (m-IFNA) in serum samples by ELISA. As shown in Fig. 4a, both wt-129 and BALB/c mice secreted significant amounts of IFNA in response to poly (I:C) stimulation.

Next, wild type 129 mice were i.p. injected with either IFNAR neutralizing antibody or mouse control IgG, at 1 day before viral inoculation and followed by two more antibody injections on 1 and 3 dpi (upper panel, Fig. 4b). Infected mice treated with IFNAR neutralizing antibody, showed a general trend of earlier onset and more severe disease manifestations, but with no statistical significance comparing to the control antibody treatment (Additional file 2: Figure S2a). In our previous study, we demonstrated that infection with EV-A71 induced limb paralysis in G129 mice (deficient in gamma-interferon) [24]. Here, in an alternative approach (lower panel, Fig. 4b), three consecutive injections with IFNG neutralizing antibody at dpi 1, 2 and 3, increased the trend of the limb paralysis rate (Additional file 2: Figure S2b). Taken together, these data suggested that both IFNAR signaling (type I IFN) and IFNG signaling (type II IFN), are protective against EV-A71 infection in an immunocompetent wt-129 mouse model.

### Blockage of IFNAR signaling leads to more severe pathology in muscle and spinal cord

We examined further the pathology of muscle and spinal cord in these EV-A71-infected mice with or without IFNAR antibody treatment. White-jaded muscle from anti-IFNAR antibody-treated mice, showed more severe fibrosis and collagen deposits (blue color) by Masson’s trichrome staining (lower panel, Fig. 4c), indicative of more severe injury and inflammation. In addition to muscle fibrosis, these treated mice showed stronger intensity of VP1-positive signals in the spinal cord, particularly in the cervical vertebrae region (Fig. 4d, Additional file 3: Figure S3). In contrast to spinal cord, we have so far detected no VP1 signal in brain sections. Altogether, IFNAR signaling does contribute to the host defense against EV-A71 infection in muscle and spinal cord.

### Type Ш IFNs cannot protect mice from EV-A71 infection

Like type I and type II IFN, type Ш IFN is known to have anti-viral activity [38, 39]. Whether type III IFN plays a role in EV-A71 infection has not been studied. To examine the potential role of type III IFN in EV-A71 infection, we treated EV-A71-infeted wt-129 mice with moue IFN-lambda (IL28a and IL28b), but detected no therapeutic efficacy (Additional file 4: Figure S4a). Moreover, we established an IL28b knockout (KO) mouse model (Materials and Methods), which was validated by genomic real-time PCR (Additional file 4: Figure S4b) and the deficiency in IFN-lambda production in the ELISA assay (Additional file 4: Figure S4c). When i.p. inoculated with EV-A71 clinical isolates (genotype B5 or C2), only one disease manifestation (1/11) was observed in this IL28b KO model (Additional file 4: Figure S4d). These data suggest that type Ш IFN does not play a major protective role in EV-A71 infection in this wt-129 model, if any.

### TLR7 agonist R837 prevented limb paralysis

**Fig. 5:**
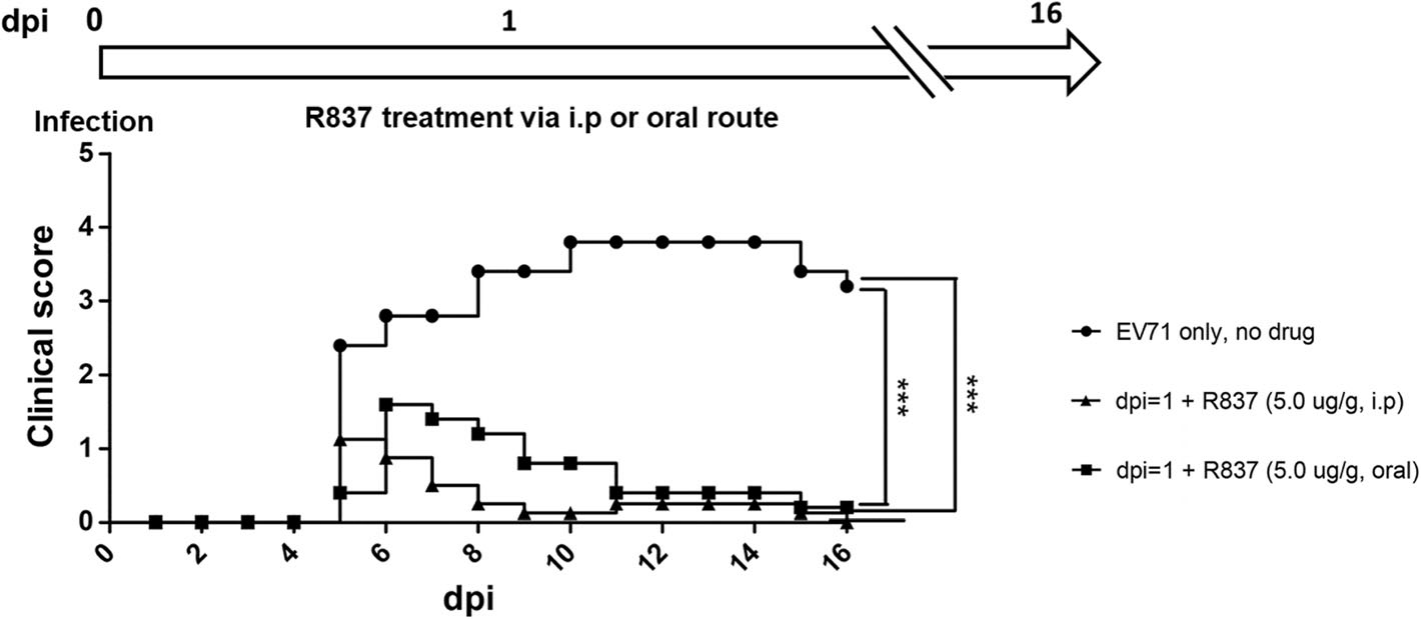
TLR7 agonist R837 rescued mice from paralysis and death efficiently after viral infection. *Upper panel:* A schematic diagram of R837 treatment via an oral or i.p. route after EV-A71 infection. *Lower panel:* Mice were treated with or without R837 (5.0 μg /g of mouse) at dpi = 1. Significant reduction in clinical score was observed only in the two groups treated with R837. *** *p* < 0.001. The average clinical scores from different experimental and control groups are plotted against dpi

Our studies described above suggest that type I and type II IFN, but not type III IFN, could reduce disease manifestation associated with EV-A71 infection. It is natural to ask if IFN-α or toll-like receptor (TLR) agonists could have any therapeutic potential for EV-A71. An FDA-approved compound R837 (also known as imiquimod) is a TLR7 agonist. It has been commonly used for genital warts caused by HPV infection [40–42]. Here, we treated the infected mice with R837 once, at the dose 5 μg/g on dpi = 1 via either an oral or an i.p. route. EV-A71-infected mice with no drug treatment usually developed early disease manifestation on 4 dpi and death between 9 and 12 dpi. R837-treated mice did not develop any limb paralysis and death up to dpi 16 (Fig. 5).

In a separate study, we tested another TLR7 agonist GS9620 (Additional file 5: Figure S5). This compound G9620 has been studied for hepatitis B treatment in a phase II clinical trial [43–45]. It was also studied in a mouse model infected with a serially-passaged mouse-adapted strain of EV-A71 [34]. Since mouse-adapted strains are known to accumulate a lot of artificial mutations not found in human natural infection, we used here instead an EV-A71 clinical isolate (non-mouse-adapted) and examined the efficacy of GS9620 in this new wt-129 mouse model.

As shown in Additional file 5: Figure S5, we i.p injected GS9620 once, at two different doses (4.5 μg/g and 3.0 μg/g), in the wt-129 mouse model at dpi = 1. Drug-treated mice infected with EV-A71 developed neither limb paralysis nor death in the entire time course (up to dpi = 21). No significant rescue of EV-A71-infected mice was observed, when drug was administered until disease onset around 4 dpi (Additional file 5: Figure S5a). In addition to the i.p route, we orally fed EV-A71-infected mice with GS9620 at dpi = 1. As shown by the clinical scores in Additional file 5: Figure S5b, we could successfully rescue the virus-infected mice by feeding GS9620 at either 4.5 μg/g or 3.0 μg/g. Although our results here suggest that GS9620 could be a therapeutic candidate for EV-A71 infection, unfortunately, we noted that treated (ip or oral, 3.0 or 4.5 μg/g) and infected wt-129 mice invariably developed very severe diarrhea at dpi = 4–5. It is therefore unclear if the “protective effect” of GS9620 is simply due to the poor retention of the input virus in the intestine with diarrhea. Intriguingly, no diarrhea was ever observed with either EV-A71 alone or drug alone.

### Timely treatment with IFN-alpha prevented paralysis and rescued hypoxia

**Fig. 6:**
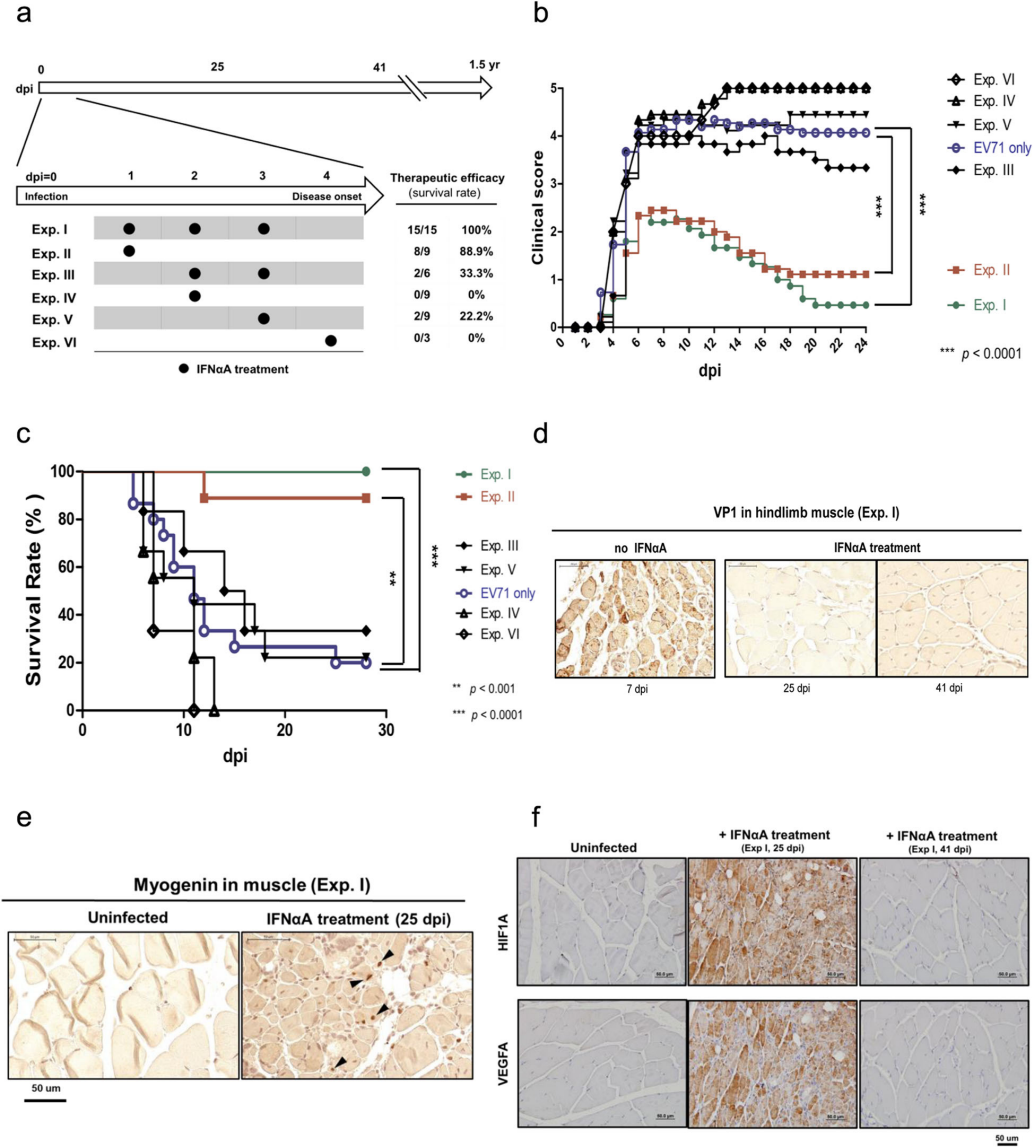
Hypoxia in EV-A71-infected muscle was rescued by timely treatment with IFNαA. **a** A schematic diagram of mouse IFNαA treatment after EV-A71 infection. Six different experimental groups of mice received mIFNαA (300 IU/g of mouse) at different dpi and frequencies of treatments. Therapeutic efficacies were assayed by survival rates. In some cases, infected and treated mice (*n* = 12) were monitored for up to 1.5 years. **b** Clinical scores were significantly reduced only in Exp. I (green) and Exp. II (red), when treated at dpi 1, or dpi 1, 2, 3. **c** Survival rates of Exp. I (green) (100%) and Exp. II (red) (88%) were significantly higher than that of the control group with no IFNαA treatment. **d** No VP1-positive signal was detected in the muscle by IHC at 25 dpi and 41 dpi in mice recovered from IFNαA treatment in Exp. 1. **e** The myogenin protein (black arrowhead) was expressed in recovered muscle via IHC staining, indicating muscle regeneration after injury. **f** HIF1A and VEGFA proteins were both expressed in newly recovered muscle (dpi = 25), but not in the fully recovered muscle (dpi = 41)

While limb paralysis and death can be rescued by timely treatment with TLR agonists, we wonder if hypoxia and viral protein can be cleared as well. In Fig. 6a, we evaluated the therapeutic efficacy of IFNαA by i.p. injection at different time points post-inoculation with EV-A71 clinical isolates. Survival rate and clinical scores were then recorded daily. The results from a total of 6 different experiments are summarized in Fig. 6a-c. In experiment I (Exp. I), all the treated mice (15/15) were prevented from paralysis. In Exp. II, near 90% of treated mice survived with no paralysis. In contrast, when IFNαA was not given within the first 24 h post-infection in Exp. III to Exp. VI, the survival rates (therapeutic efficacies) dropped down to 33, 0, 22, and 0%, respectively. These data revealed that IFNαA treatment should be given as early as possible upon exposure to EV-A71 infection. In those mice receiving timely treatment with IFNαA, we detected no VP1 protein expression in the hindlimb muscle via IHC at dpi 25 and dpi 41 (Fig. 6d). Using an anti-myogenin antibody for IHC staining, we detected myogenin-positive signals in the muscle of IFNαA-treated wt-129 mice at 25 dpi, indicating an ongoing residual muscle regeneration after clearance of VP1 (Fig. 6d and e). We also examined the hypoxia status in muscle sections from the recovering (dpi = 25) and the fully-recovered (dpi = 41) mice by IHC staining with anti-HIF1A and anti-VEGFA antibodies (Fig. 6f). Despite the lack of detectable VP1 in muscle sections at 25 dpi, HIF1A- and VEGFA-positive signals remained detectable in the recovering mice at 25 dpi. However, they were no longer detectable in fully-recovered muscles at 41 dpi. In summary, these data strongly suggest that TLR7 agonists and IFNαA could be considered for clinical trials in the next EV-A71 outbreaks in endemic areas.

## Discussion

EV-A71 has a narrow host range, and clinical isolates of human EV-A71 cannot infect rodent cells efficiently without prior adaptation via serial passages. However, mouse-adapted variants of EV-A71 are known to accumulate a lot of artificial mutations, which cannot be found in patients in natural infection [46]. In human receptor hSCARB2 transgenic mouse models, EV-A71 can replicate in vivo and cause paralysis and death [19, 20]. It is puzzling that this wt-129 mouse model contains no transgenic human receptor for viral entry. In addition to entry receptors, immune system also played an important role in the susceptibility to viral infection [21, 24–26]. Intriguingly, this wt-129 mouse model is neither immunodeficient nor transgenic for any human receptors. One possible explanation for this puzzle is that alternative entry receptors could be available in wt-129 mice, such as PSGL-1, annexin II and nucleolin [16–18]. These putative mouse receptors may support cross-species infection with EV-A71 clinical isolates at a lower efficiency. While the exact mechanism for viral entry in this wt-129 model remains unclear, there is no doubt that it is an immunocompetent and user-friendly model with no genetic modification in the mouse genome.

Serendipitously, we encountered a novel “white-jade” phenotype in EV-A71-infected wt-129 mice. This phenotype is characteristic in its sporadic pale color patches in the pink background of hindlimb muscle with myositis (Fig. 2a). In one report in literature, the mice infected with an adapted strain of EV-A71 developed necrotizing myositis of the respiratory muscle, and caused severe hypoventilation by monitoring respiratory functions [47]. In this mouse model, the systemic hypoxia is caused indirectly by the failure in the smooth muscle involved in lung respiration. In human clinical studies, hypoxia has been noted by different research groups [48–51]. For example, hypoxia is one of the factors correlated with long term sequelae in Taiwanese pateints [48, 49]. In addition, both hypoxia ischemia of grey nuclei and acute progressive hypoxia were observed in clinical manifestations [50, 51]. In our EV-A71 infected wt-129 mice, hypoxia occurred locally and directly in the white-jade patches of the hindlimb skeletal muscle, without involving any malfunction in the lung or respiration. We did not see any difference in lung sections between infected and non-infected wt-129 via H & E staining. Nor was VP1 positive signal detected in the lung in EV-A71-infected mice. Although muscle pathology has not been reported in EV-A71 clinical literature, it is noteworthy whether muscle infection with EV-A71 can be detected in patients. Although EV-A71 is exclusively neurotropic in the stat-1 KO mice [24], muscle infection with EV-A71 is very common in the vast majority of the EV-A71 mouse models [1]. In the case of poliovirus, muscle is an intermediate reservoir for the spreading from muscle to CNS [52]. In addition, spleen atrophy has been documented in autopsied samples from EV-A71 patients [53, 54].

It is known that ischemia tissue can be identified by its lack of mitochondria activity using the TTC (triphenyltetrazolium chloride) staining [55]. By using this TTC staining, we seemed to detect positive signal of ischemia only in the white-jaded muscle, but not in the non-white-jaded muscle (data not shown). Unfortunately, this interpretation of the TTC data here is less certain, since the readout of a positive TTC staining signal is white in color, which cannot stand out from the white background of the white-jaded muscle.

One paradox here is that, despite the high level of VEGF in the white-jaded muscle, its white color suggests an apparent lack of red blood cells and angiogenesis. There is no indication of anemia in the circulating red blood cells (Fig. 2d). Instead, we found increased levels of neutrophils and monocytes, supporting a certain degree of inflammation after EV-A71 infection. Two transcription factor binding sites have been identified in the VEGFA promoter. One can bind with HIF1A, and the other can be recognized by activated NF-κB [56]. NF-κB can be activated by pro-inflammatory cytokines (e.g. TNF-α) or TLR signals during inflammation [57]. It is therefore possible that the high level expression of VEGF in the non-white-jaded muscle (Additional file 1: Figure S1c & d) could originate from the NF-κB, rather than HIF1A, signaling.

In our current study, we used IFNAR-specific neutralizing antibody to repress IFN signaling in the wt-129 model (Fig. 4). We observed earlier disease onset, more severe disease outcome and fibrosis of the hindlimb muscle (Fig. 4c). Similarly, in the spinal cord sections, VP1 protein appeared to be more widespread and stronger in intensity upon treatment with IFNAR-specific neutralizing antibody (Fig. 4d and Additional file 3: Figure S3). In contrast, we detected no VP1 signal in the brain, with or without treatment with anti-IFNAR antibody. At present, it remains unclear why VP1 is detectable in the spinal cord, but undetectable in the brain. Nevertheless, the results seem to suggest that EV-A71 does not reach spinal cord by passing through the blood-brain barrier, at least in this wt-129 mouse model.

Previously, prophylactic treatment or co-treatment with IFN or poly-IC could protect mice infected with a serially-passaged mouse-adapted strain of EV-A71 [58]. We asked here whether activation of IFN signaling, *after* virus inoculation, can have any therapeutic potential in this wt-129 mouse model infected with non-mouse-adapted clinical isolates? We were encouraged that timely treatments via an i.p. route with mouse IFNαA, or via an oral route with a new TLR7 agonist R837, achieved very high protection efficacies. Unlike GS-9620, we observed no side effect of severe diarrhea with R837. Recently, 30% of EV-A71-infected BALB/c mice can be protected from death by injecting adenovirus expressing mouse IFNA within 12 h post-inoculation with virus [59]. In clinical studies, when HFMD patients with mild symptoms were injected with human IFNα1b, the treatment group appeared to recover sooner, in terms of fever and viral load, than the placebo group [60, 61]. Instead of using the injection route, more convenient oral medicine is needed for broad and effective protection of children from EV-A71 infection. In this regard, TLR7 agonists, such as R837, could be a promising example of oral medicine for future therapeutic trials. Finally, it also warrants further investigation on the therapeutic potential of anti-hypoxia drugs in EV-A71 infection.

While hypoxia is shown to be involved in EV-A71 pathogenesis in spleen and muscle (Figs. 2 and 3), we speculate here that it could represent a more general pathogenesis mechanism, e.g., involved in the CNS infection in children and mouse models [8, 9, 24, 26] . Most recently, EV-A71 was shown to be able to directly attack the cardiopulmonary system via oral infection in a NOD/SCID mouse model [25]. A closer examination of the infected hearts revealed VP1-positive staining, leukocyte infiltration, severe cardiomyocyte apoptosis, high frequency abnormal EKG, and several different waves of inflammatory cytokines during the course of infection and disease manifestations. Similarly, infiltrated leukocytes and abundant M2 type macrophage were identified in the orally infected lung. Overall, it is possible that hypoxia could also be involved in the pathogenesis of other infected organs, such as brain, heart and lung.

## Conclusions

To investigate EV-A71 pathogenesis and treatments, we established a new mouse strain 129 model for research. This user-friendly immunocompetent mouse model could offer a robust platform for future research on EV-A71 and other emerging infectious diseases. Hypoxia is an important biological phenomenon when oxygen supply is in shortage. We demonstrated here that both hypoxia and limb paralysis can be successfully rescued at a high efficacy by timely treatment with immune-modulating drugs.

## Supplementary information

**Additional file 1: Figure S1.** Comparisons between white-jaded and non-white-jaded muscles. **a & b** Paraffin-embedded muscle sections were stained with various antibodies specific for lymphocyte and macrophage markers. Massive infiltration of M1 macrophage (CD68) was detected in both white-jaded and non-white-jaded muscles. **c** Infectious virus was recovered and titrated from EV-A71-infected muscle. No significant difference was found between white-jaded and non-white-jaded muscle. **d** Analysis by complete blood count (CBC) revealed no statistically significant difference in the total numbers of red blood cells (RBC), hemoglobin concentration (HGB value) and hematocrit (HCT value) between uninfected (*N* = 3) and EV-A71-infected (*N* = 6) mice. In contrast to the significant decrease of lymphocytes in EV-A71-infected mice, both neutrophil (NEU) and monocyte (MONO) were increased significantly in EV-A71 infected mice. **e** Strong VEGFA expression was detected by IHC in both white-jaded and non-white-jaded muscles. **f** Similar levels of VEGFA protein expression were detected by ELISA between white-jaded and non-white-jaded muscles. ns, not statistically significant. **g** A significantly increased level of VEGFA in the blood circulation was detected by ELISA in EV-A71-infected mice (6–9 dpi) with disease manifestation. No increase in VEGF was detected in uninfected control mice. Dotted line represents the detection sensitivity in this ELISA kit.

**Additional file 2: Figure S2.** Inhibition of IFN signaling enhanced EV-A71 infection and pathogenesis. **a** When treated with IFNAR neutralizing antibody, a trend of more severe paralysis rate was observed. However, no statistical significance was noted between the experimental group treated with neutralizing antibody and the control group treated with mouse IgG antibody. **b** IFNG (interferon-gamma) antibody was administered on 1, 2, and 3 dpi. Although a trend of more severe paralysis rate was observed, no statistical significance was noted between the experimental group and the control group.

**Additional file 3: Figure S3.** Treatment with neutralizing antibody against IFNAR enhanced VP1 signals in the thoracic spinal cord. Thoracic vertebrae can be divided into 4 segments. In control mice without IFNAR antibody treatment, VP1 protein signals in the spinal cord were weak, and limited to only a small segment 2 of the thoracic vertebrae. In contrast, in mice treated with anti-IFNAR antibody, VP1 protein signals in thoracic segment 2 were stronger, and more wide-spread to other segments of cervical vertebrae (Fig. 4d).

**Additional file 4: Figure S4.** Mouse type Ш IFNs did not protect EV-A71-infected wt-129 mice. **a** One-week-old wt-129 mice were infected with EV-A71 and treated with three shots of IFNλ (IL28a or IL28b) at different doses on dpi 1, 2 and 3. All mice were monitored daily for survival curve. There was no detectable protective effect from IFNλ treatment. **b** Genomic DNA was extracted from IL28b genetically modified mice (Materials and Methods), and RT-qPCR was used to distinguish between wild type, heterozygote and homozygote knockout mice. Unlike DNA samples from WT and heterozygote IL-28b (+/−), no PCR signal can be amplified from the DNA sample of the IL28b KO mice.** c** Unlike the WT B6N mice, IL28b KO mice (lineage clone 1 and clone 2) produced only IFNα (right panel), but no IFNλ (left panel), by poly I:C stimulation. Wild type C57BL/6 N and 3-week-old KO mice were injected with high MW poly I:C via an i.p. route. Sera were collected 4 h post-injection, and both IFNλ and IFNα were measured by the ELISA assay. **d** One-week-old IL28b KO mice were infected with different genotypes of EV-A71 (gt B5 and C2). Only one out of eleven (1/11) developed paralysis and death. Wild type B6 mice were resistant to EV-A71 infection (0/6). In contrast, the hybrid mice, containing the hSCARB2 receptor transgene and the stat-1 knockout background, were highly susceptible to EV-A71 infection and pathogenesis (10/10).

**Additional file 5: Figure S5.** Oral intake or ip injection with a TLR7 agonist GS-9620 after virus inoculation efficiently rescued mice from paralysis and death. a *Upper panel:* A schematic diagram of GS-9620 treatment via an i.p. route after EV-A71 infection. Mice of different experimental groups received one shot of GS-9620 at dpi = 1 or dpi = 4 (disease onset). *Lower panel:* Clinical scores were significantly lower in mice receiving timely treatment of GS-9620 at dpi = 1. Red, EV-A71-infected mice with no drug treatment; Blue, 4.5 μg GS-9620 per g of body weight at dpi = 1; Green, 3.0 μg GS-9620 per g of body weight at dpi = 1. **b** *Upper panel:* A schematic diagram of GS-9620 treatment via an oral route after EV-A71 infection. *Lower panel:* Three experimental groups of mice received GS-9620 at three different doses (0, 3.0 and 4.5 μg /g of mouse) at dpi = 1. Significant reduction in clinical score was observed in the groups treated with GS-9620.

**Additional file 6: Table S1.** Primer sequences for detecting the diagnostic PCR Products by Taqman assay.

## Data Availability

All data generated in this study are included in this manuscript.

## Abbreviations

CBC: Complete blood count
CNS: Central nerve system
EV-A71 or EV-A71: Enterovirus 71
HCT: Hematocrit
HFMD: Hand-foot-and-mouth disease
HGB: Hemoglobin concentration
HIF-1: Hypoxia-inducible factor 1
hPSGL-1: Human P-selectin glycoprotein ligand-1
hSCARB2: Human scavenger receptor class B
i.p. route: Intraperitoneal route
IFNA: Alpha-interferon
IFNG: Gamma-interferon
IHC staining: Immunohistochemical staining
KO: Knockout
Poly (I:C)-HMW: Poly (I:C) with high molecular weight
Poly (I:C)-LMW: Poly (I:C) with low molecular weight
RD: Rhabdomyosacroma
TLR: Toll-like receptor
TTC: Triphenyltetrazolium chloride
VEGF: Vascular endothelial growth factor
wt-129: 129S2/SvPasCrlBltw
