# Supplementary figures and images for: Hypoxia and therapeutic treatment of EV-A71 with an immune modulator TLR7 agonist in a new immunocompetent mouse model
Source: J Biomed Sci. 2019 Nov 11;26:93. doi: 10.1186/s12929-019-0585-y (PMC6849267; doi:10.1186/s12929-019-0585-y)

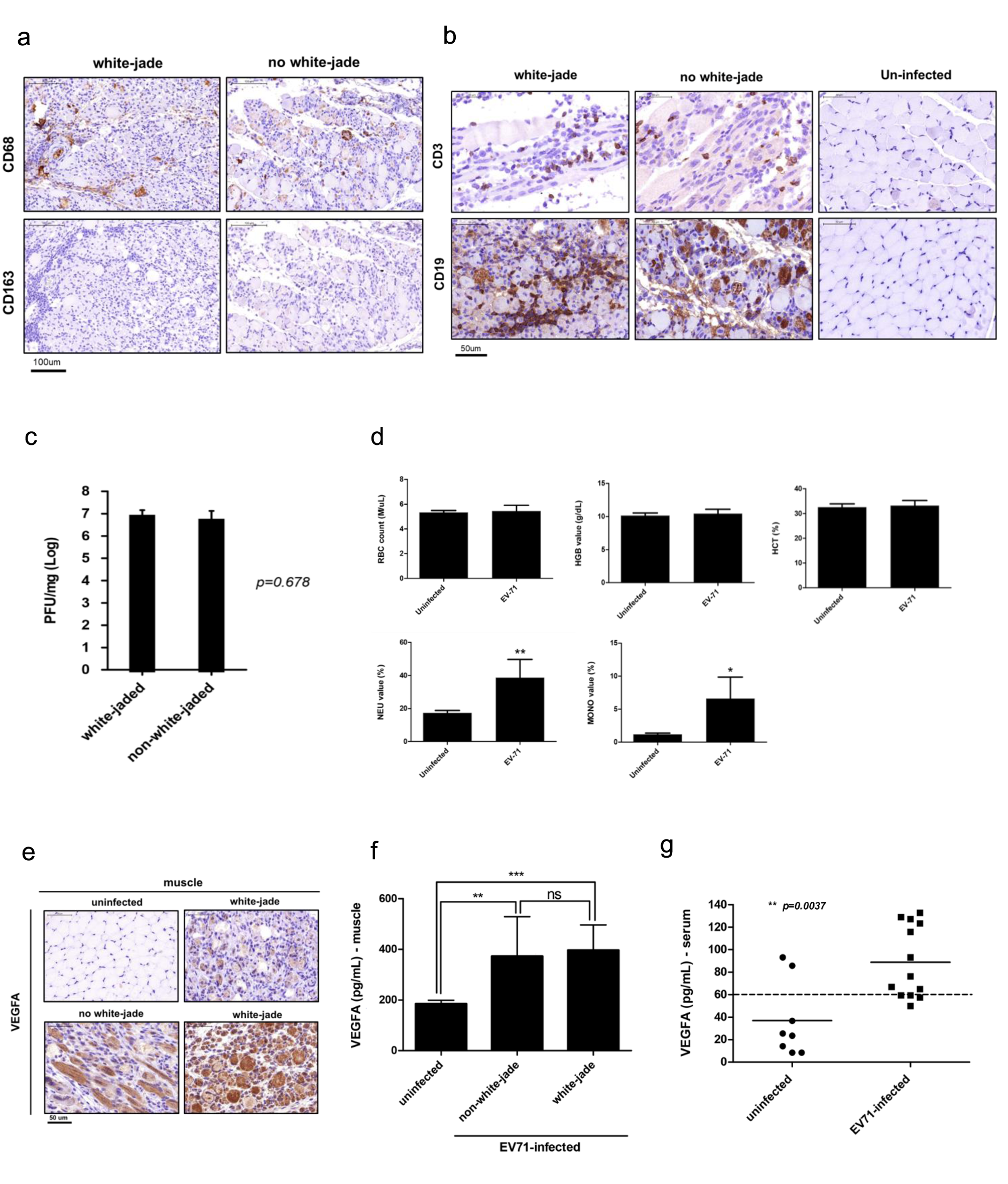

Supplement: Supplementary file 1 — Additional file 1: Figure S1. Comparisons between white-jaded and non-white-jaded muscles. a & b Paraffin-embedded muscle sections were stained with various antibodies specific for lymphocyte and macrophage markers. Massive infiltration of M1 macrophage (CD68) was detected in both white-jaded and non-white-jaded muscles. c Infectious virus was recovered and titrated from EV-A71-infected muscle. No significant difference was found between white-jaded and non-white-jaded muscle. d Analysis by complete blood count (CBC) revealed no statistically significant difference in the total numbers of red blood cells (RBC), hemoglobin concentration (HGB value) and hematocrit (HCT value) between uninfected (N = 3) and EV-A71-infected (N = 6) mice. In contrast to the significant decrease of lymphocytes in EV-A71-infected mice, both neutrophil (NEU) and monocyte (MONO) were increased significantly in EV-A71 infected mice. e Strong VEGFA expression was detected by IHC in both white-jaded and non-white-jaded muscles. f Similar levels of VEGFA protein expression were detected by ELISA between white-jaded and non-white-jaded muscles. ns, not statistically significant. g A significantly increased level of VEGFA in the blood circulation was detected by ELISA in EV-A71-infected mice (6–9 dpi) with disease manifestation. No increase in VEGF was detected in uninfected control mice. Dotted line represents the detection sensitivity in this ELISA kit. [file 12929_2019_585_MOESM1_ESM.tif]

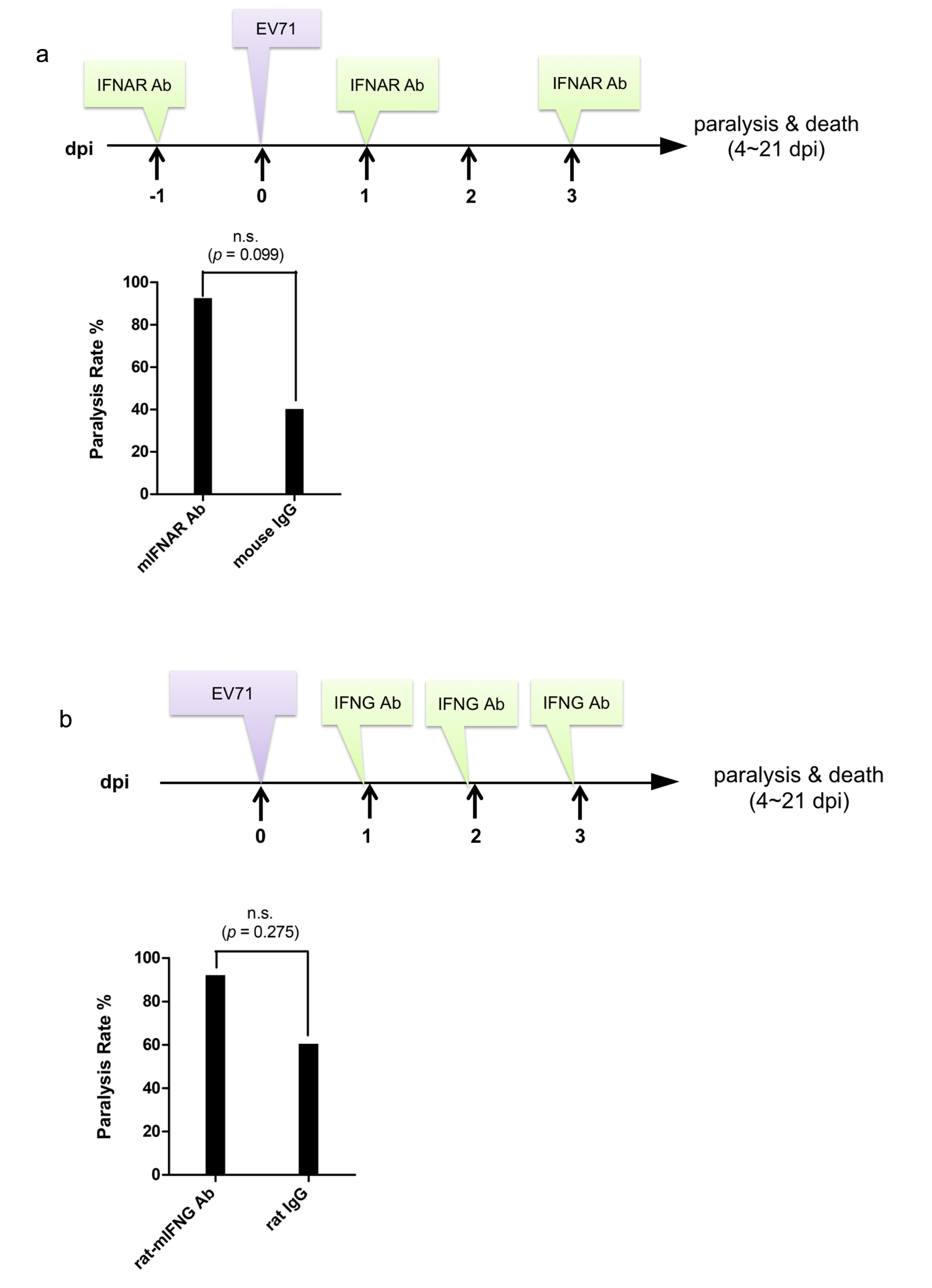

Supplement: Supplementary file 2 — Additional file 2: Figure S2. Inhibition of IFN signaling enhanced EV-A71 infection and pathogenesis. a When treated with IFNAR neutralizing antibody, a trend of more severe paralysis rate was observed. However, no statistical significance was noted between the experimental group treated with neutralizing antibody and the control group treated with mouse IgG antibody. b IFNG (interferon-gamma) antibody was administered on 1, 2, and 3 dpi. Although a trend of more severe paralysis rate was observed, no statistical significance was noted between the experimental group and the control group. [file 12929_2019_585_MOESM2_ESM.tif]

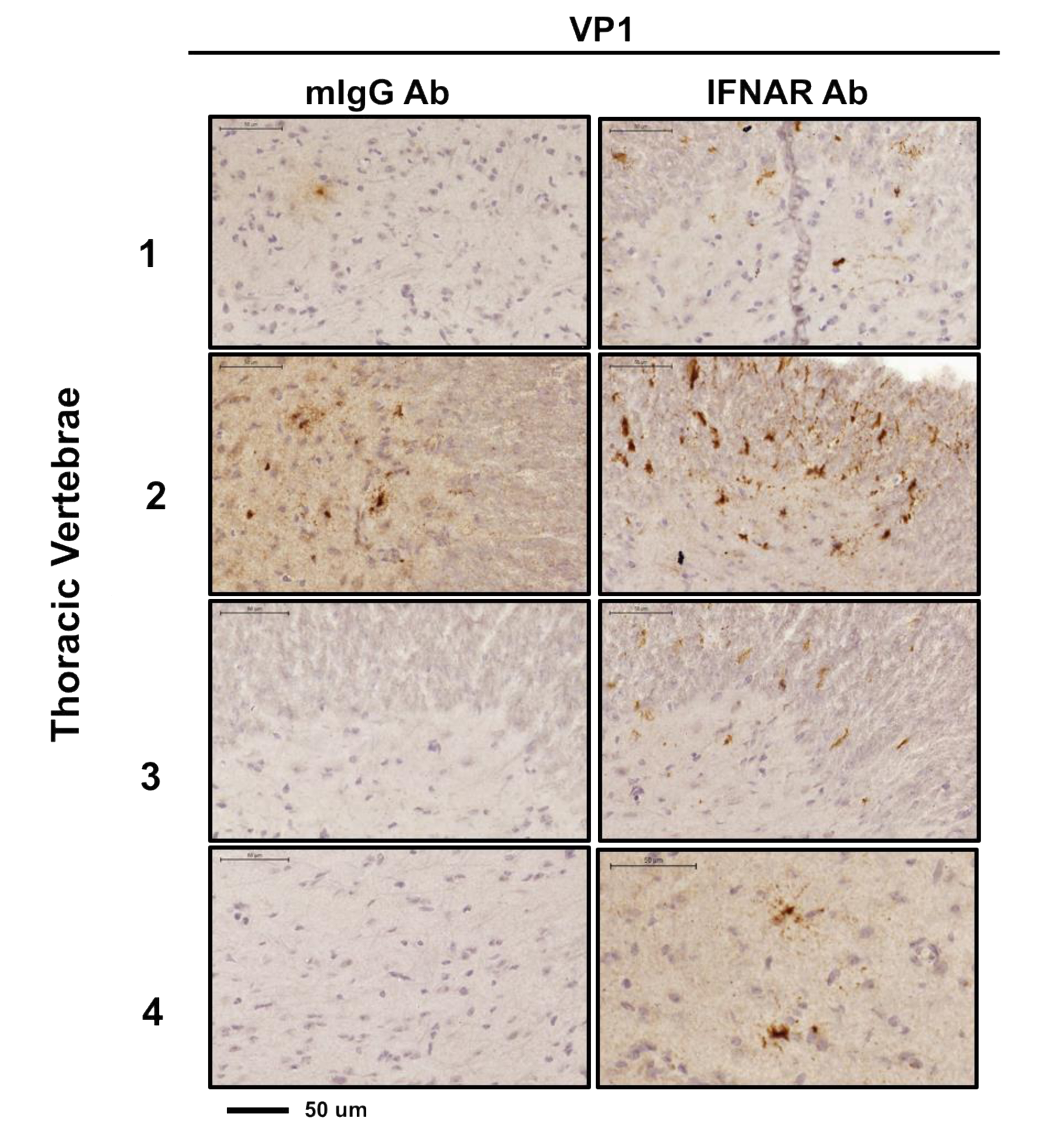

Supplement: Supplementary file 3 — Additional file 3: Figure S3. Treatment with neutralizing antibody against IFNAR enhanced VP1 signals in the thoracic spinal cord. Thoracic vertebrae can be divided into 4 segments. In control mice without IFNAR antibody treatment, VP1 protein signals in the spinal cord were weak, and limited to only a small segment 2 of the thoracic vertebrae. In contrast, in mice treated with anti-IFNAR antibody, VP1 protein signals in thoracic segment 2 were stronger, and more wide-spread to other segments of cervical vertebrae (Fig. 4d). [file 12929_2019_585_MOESM3_ESM.tif]

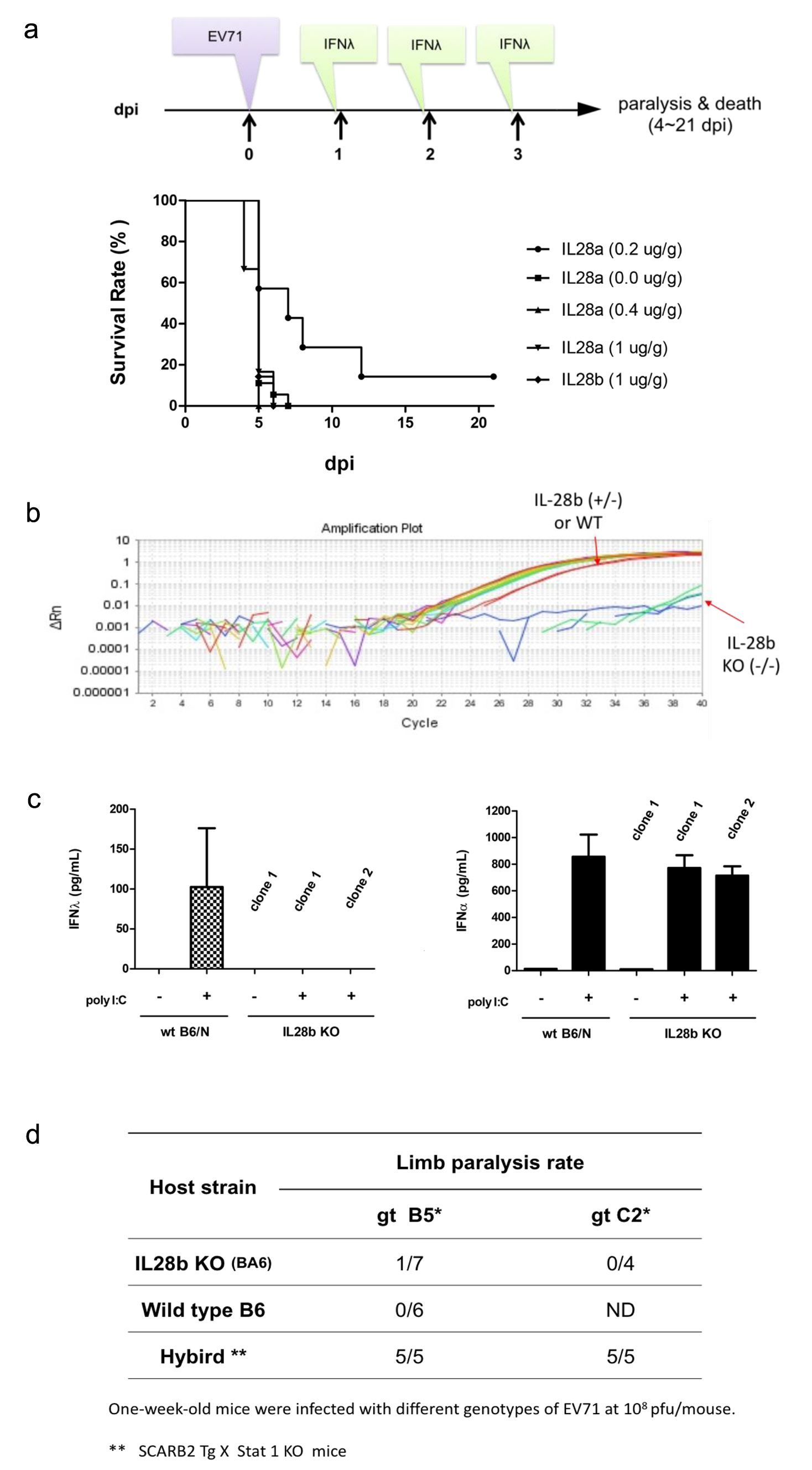

Supplement: Supplementary file 4 — Additional file 4: Figure S4. Mouse type Ш IFNs did not protect EV-A71-infected wt-129 mice. a One-week-old wt-129 mice were infected with EV-A71 and treated with three shots of IFNλ (IL28a or IL28b) at different doses on dpi 1, 2 and 3. All mice were monitored daily for survival curve. There was no detectable protective effect from IFNλ treatment. b Genomic DNA was extracted from IL28b genetically modified mice (Materials and Methods), and RT-qPCR was used to distinguish between wild type, heterozygote and homozygote knockout mice. Unlike DNA samples from WT and heterozygote IL-28b (+/−), no PCR signal can be amplified from the DNA sample of the IL28b KO mice. c Unlike the WT B6N mice, IL28b KO mice (lineage clone 1 and clone 2) produced only IFNα (right panel), but no IFNλ (left panel), by poly I:C stimulation. Wild type C57BL/6 N and 3-week-old KO mice were injected with high MW poly I:C via an i.p. route. Sera were collected 4 h post-injection, and both IFNλ and IFNα were measured by the ELISA assay. d One-week-old IL28b KO mice were infected with different genotypes of EV-A71 (gt B5 and C2). Only one out of eleven (1/11) developed paralysis and death. Wild type B6 mice were resistant to EV-A71 infection (0/6). In contrast, the hybrid mice, containing the hSCARB2 receptor transgene and the stat-1 knockout background, were highly susceptible to EV-A71 infection and pathogenesis (10/10). [file 12929_2019_585_MOESM4_ESM.tif]

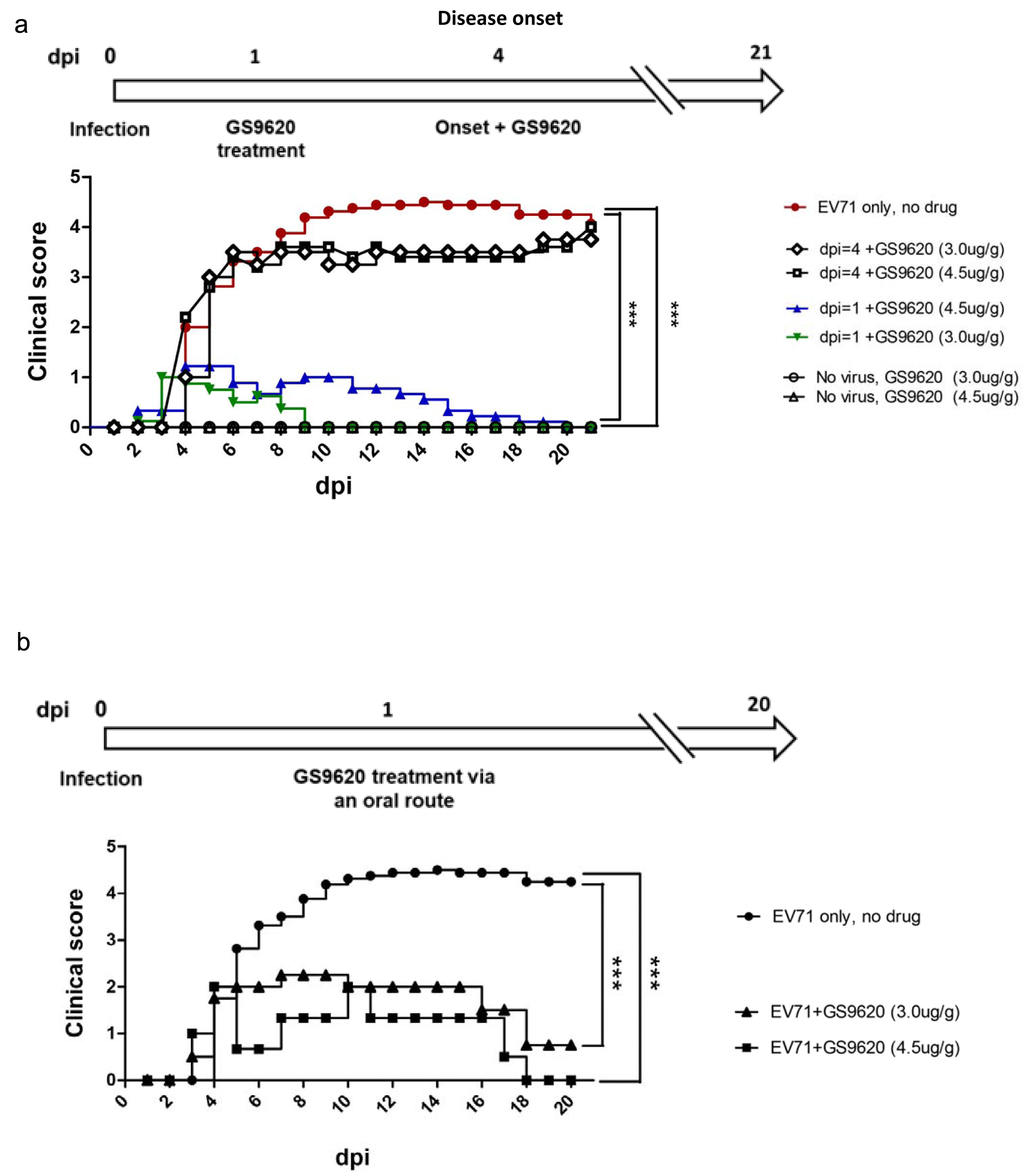

Supplement: Supplementary file 5 — Additional file 5: Figure S5. Oral intake or ip injection with a TLR7 agonist GS-9620 after virus inoculation efficiently rescued mice from paralysis and death. a Upper panel: A schematic diagram of GS-9620 treatment via an i.p. route after EV-A71 infection. Mice of different experimental groups received one shot of GS-9620 at dpi = 1 or dpi = 4 (disease onset). Lower panel: Clinical scores were significantly lower in mice receiving timely treatment of GS-9620 at dpi = 1. Red, EV-A71-infected mice with no drug treatment; Blue, 4.5 μg GS-9620 per g of body weight at dpi = 1; Green, 3.0 μg GS-9620 per g of body weight at dpi = 1. b Upper panel: A schematic diagram of GS-9620 treatment via an oral route after EV-A71 infection. Lower panel: Three experimental groups of mice received GS-9620 at three different doses (0, 3.0 and 4.5 μg /g of mouse) at dpi = 1. Significant reduction in clinical score was observed in the groups treated with GS-9620. [file 12929_2019_585_MOESM5_ESM.tif]

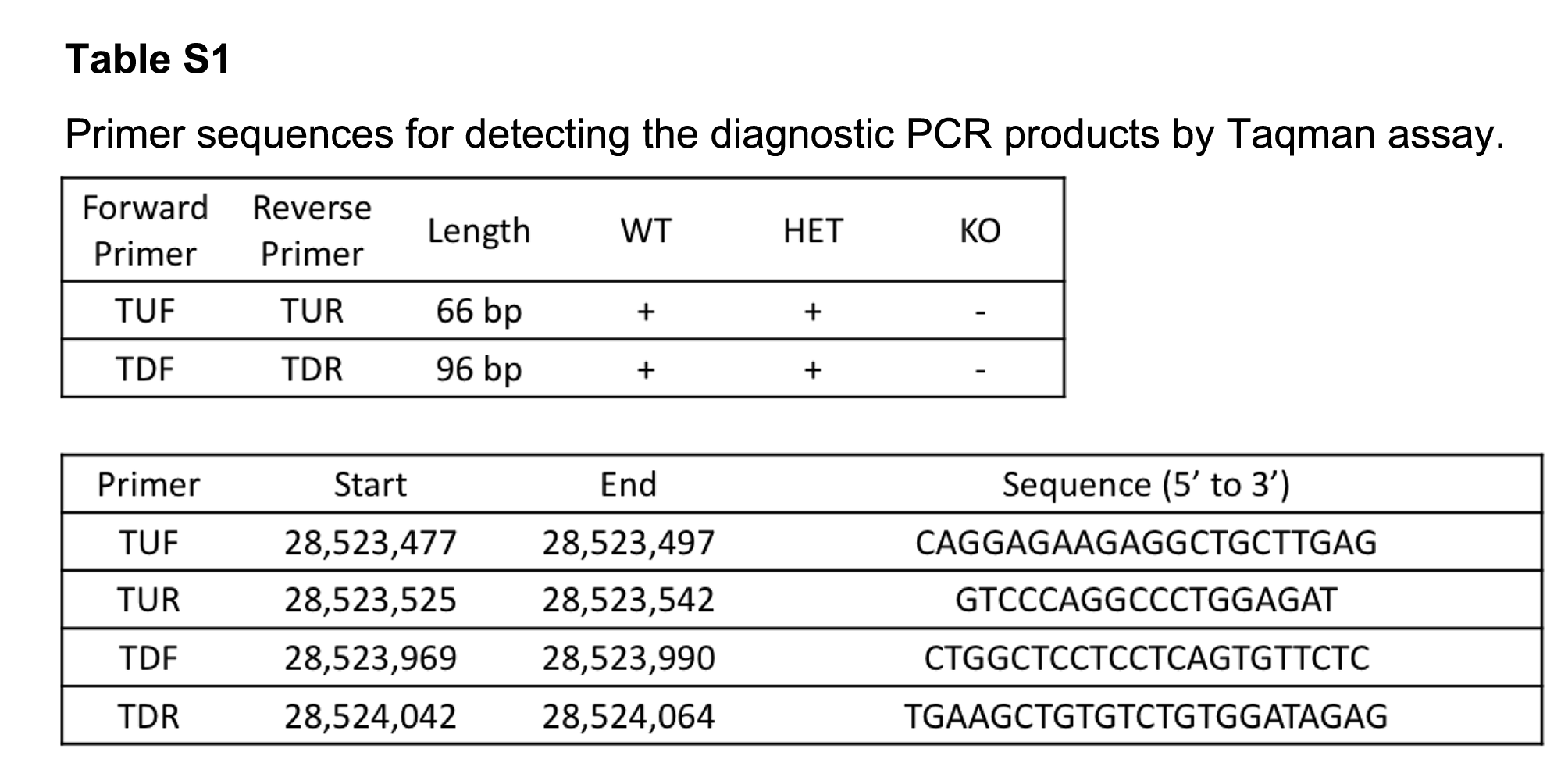

Supplement: Supplementary file 6 — Additional file 6: Table S1. Primer sequences for detecting the diagnostic PCR Products by Taqman assay. [file 12929_2019_585_MOESM6_ESM.tif]
